# Assessment of Creep Reduction Factors of High-Density Polyethylene Geogrids Using Conventional and Stepped Isothermal Methods

**DOI:** 10.3390/ma19040714

**Published:** 2026-02-12

**Authors:** Hang-Won Cho, Kap-Jin Kim, Nigel Edwin Wrigley, Hyun-Jin Koo, Suk-Won Choi

**Affiliations:** 1 Department of Materials Science and Engineering, Kyung Hee University, 1732 Deogyeong-daero, Yongin 17104, Republic of Korea; hwcho@fiti.re.kr (H.-W.C.); kjkim@khu.ac.kr (K.-J.K.); 2 FITI Testing & Research Institute, Magokjungang 8-ro 3-gil, Seoul 07791, Republic of Korea; 3 NEW Associates, Flat 11 Witley, 387 Sandbanks Road, Poole BH14 8HR, UK; nigel@newassociates.com

**Keywords:** time–temperature superposition, stepped isothermal method, block shift analysis, high density polyethylene, geosynthetics, creep reduction factor

## Abstract

The long-term creep performance of geosynthetics is crucial for the safe design of reinforced-soil structures. Previous studies have not sufficiently clarified the long-term creep behavior of high-density polyethylene (HDPE) geogrids or the influence of different failure criteria. Therefore, further research is needed to validate creep reduction factors’ (RF_CR_) estimation and the applicability of the stepped isothermal method (SIM). In this study, the creep behavior of HDPE geogrids was examined using both conventional creep tests and SIM, conducted in accordance with ISO 13431 and ASTM D6992. Master curves were generated under load levels representing 40–60% of the ultimate tensile strength. The SIM results matched with the conventional tests in the early stage but exhibited higher creep strains beyond 1000 h, primarily due to the thermal sensitivity of HDPE. RF_CR_ values were determined using two design criteria, namely, 20% creep strain and creep rupture. For a 100-year design life, the RF_CR_ values based on a 20% creep strain were determined to be 3.04 and 2.43 based on the combined data and block-shift analysis, respectively, whereas the rupture criterion yielded a lower value of 2.30. These findings demonstrate that the 20% strain limit provides a more conservative and reliable criterion for estimating the long-term design strength. This study confirms the applicability of SIM for accelerated creep evaluation and provides practical guidance for the selection of RF_CR_ values in reinforced-soil design.

## 1. Introduction

The long-term creep behavior of geosynthetics is a critical factor governing the stability and serviceability of geosynthetic reinforced-soil (GRS) structures, as excessive time-dependent deformation or creep rupture may lead to progressive wall deformation or even structural failure [1,2,3,4,5]. Geogrids manufactured from high-density polyethylene (HDPE), polypropylene (PP), polyethylene terephthalate (PET), and polyvinyl alcohol (PVA) exhibit distinct viscoelastic and nonlinear viscoelastic responses governed by their microstructure and thermal sensitivity [6,7,8,9]. Accurate prediction of long-term creep performance and determination of appropriate creep reduction factors (RF_CR_) are therefore essential in design practice.

Since the 1980s, extensive studies have investigated the creep behavior of HDPE geogrids through conventional long-term testing, time–temperature superposition (TTS), and accelerated approaches [10,11,12,13,14,15]. HDPE is particularly susceptible to temperature-induced creep acceleration due to its nonlinear viscoelasticity, especially near secondary creep regime [16,17,18,19,20]. Polymer studies have identified lamellar slip, amorphous-phase mobility, chain disentanglement, and microstructural evolution under load as key mechanisms contributing to creep deformation [21,22,23].

The stepped isothermal method (SIM), standardized in ASTM D6992 [24], has become a widely adopted accelerated testing technique for predicting creep deformation and rupture in geosynthetics. While SIM has been validated for PET geogrids [25,26,27,28,29,30,31,32,33,34], discrepancies between SIM and conventional creep curves have been reported for HDPE, attributed to accumulated thermal strain and nonlinear viscoelastic effects [13,31,34,35]. In addition to SIM, accelerated stress relaxation techniques have also been applied to HDPE geogrids, providing alternative pathways for long-term creep prediction. These findings highlight the need for a systematic, side-by-side comparison of SIM, conventional creep testing, and other accelerated approaches specifically for HDPE geogrids.

Different criteria have also been proposed to define creep failure. Early design approaches emphasized excessive creep strain as the governing factor for long-term performance in reinforced-soil structures. Zornberg et al. introduced a deviation-from-linearity criterion, and several design codes recommend strain-based limits between 1% and 10%, depending on structure type [1,2,3,33]. Full-scale field monitoring also supports strain-limited criteria for conservative and reliable design. ISO TS 20432 [36] currently adopts a rupture-based definition of creep failure for RF_CR_ calculation.

Analytical approaches based on TTS and block shifting have further been employed to reduce test duration and construct master curves [13,37,38]. However, their accuracy depends on shift factors, which may not be strictly applicable to HDPE due to its nonlinear viscoelastic properties [16,21,37]. Consistency among SIM, conventional creep testing, stress–relaxation-based accelerated methods, and block-shift-based TTS must be evaluated for reliable RF_CR_ calculation.

Despite extensive research on creep behavior, several important knowledge gaps remain, particularly for HDPE geogrids. First, although inconsistencies between SIM and conventional long-term creep responses have been reported, these findings are fragmented and have not been systematically evaluated through a controlled, side-by-side comparison using the same material and load conditions. Second, the influence of different failure criteria—strain-based versus rupture-based—on RF_CR_ estimation has not been thoroughly clarified, even though excessive creep strain is often the dominant serviceability concern in reinforced-soil structures. Third, while TTS-based block shifting is widely used for long-term creep prediction by shifting short-term test blocks, its applicability to HDPE—whose nonlinear viscoelastic behavior challenges the assumptions of parallelism and linear shift factors—has not been rigorously validated.

To address these gaps, this study develops a unified creep evaluation framework for HDPE geogrids that integrates (i) conventional long-term creep tests, (ii) accelerated SIM testing, and (iii) TTS-based block-shift analysis. This multi-method comparison enables direct assessment of methodological consistency across test approaches. Furthermore, RF_CR_ values are quantified using two distinct failure criteria—20% creep strain and creep rupture—to explicitly evaluate the impact of failure definition on long-term design recommendations. By combining experimental, accelerated, and analytical approaches within a single comprehensive investigation, this study provides practical guidance for design codes and enhances the reliability of long-term creep prediction and RF_CR_ selection for HDPE geogrids.

In this study, the creep behavior of HDPE geogrids was evaluated using both conventional creep tests (ISO 13431) [39], SIM (ASTM D6992), and block-shift analysis. Master curves were compared to assess agreement across loading and temperature conditions. RF_CR_ values were determined using two criteria—20% creep strain and creep rupture—and block-shift analysis was applied to evaluate long-term predictions. This integrated approach provides a comprehensive assessment of creep behavior and RF_CR_ determination for HDPE geogrids.

## 2. Materials and Methods

### 2.1. Materials

**Figure 1:**
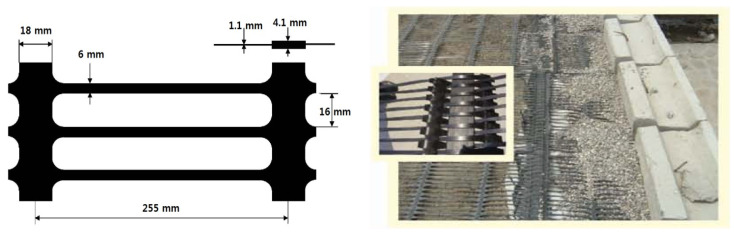
Photographs of the HDPE geogrid specimen: (**left**) laboratory specimen showing its dimensions, and (**right**) field installation of the HDPE geogrid in a reinforced-soil retaining wall.

A uniaxial geogrid composed of HDPE, which is commonly used for the reinforcement of retaining walls and slopes, was selected for this study. All specimens were prepared from a single roll of the same lot to ensure consistency. In accordance with ISO 10319 [40], each specimen was made to include one geogrid node at the center, with a width of 20 cm, and these were used for both conventional creep and SIM testing. Figure 1 presents a photograph of HDPE geogrid specimen, illustrating its dimensions and typical configuration. The tensile properties in the machine direction were determined in accordance with ISO 10319 and are summarized in Table 1. The geogrid exhibited an UTS of 95.2 kN/m and an average strain at peak load of 10.8% under standard conditions of (20 ± 2) °C and (65 ± 5)% relative humidity. A commercially available HDPE geogrid was divided into two sets for a comparative evaluation using SIM and conventional creep tests.

### 2.2. Conventional Creep Tests

**Figure 2:**
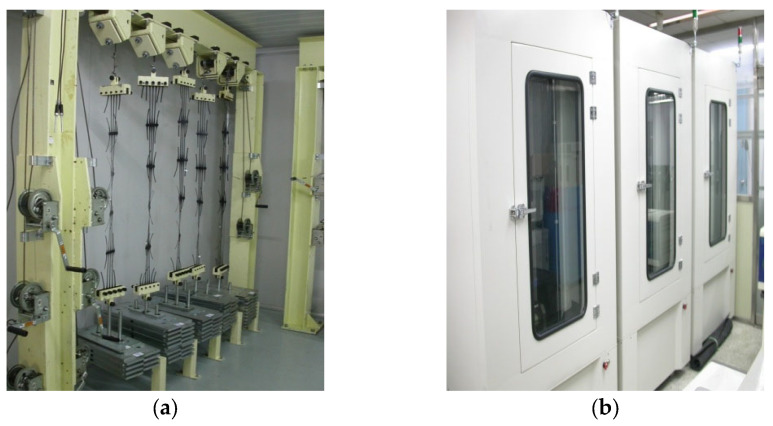
Photographic images showing the equipment employed to perform the (**a**) conventional creep and (**b**) SIM tests for the HDPE geogrids.

The conventional creep tests were conducted in accordance with ISO 13431. The conventional creep test equipment is illustrated in Figure 2a. Each specimen was loaded under a constant tensile load while being maintained in temperature-controlled chambers set at 20, 30, 40, and 50 °C (±1 °C). A total of 75 test stations were used, and each specimen had a gauge length of approximately 750 mm. For each test temperature, multiple specimens were subjected to creep loads corresponding to 40–60% of the ultimate tensile strength (UTS). Creep strain was recorded continuously or at fixed intervals until one of the following conditions was reached: (i) the specimen achieved 20% creep strain, or (ii) creep rupture occurred. Because the temperature remained constant throughout each individual test, the conventional creep method provides true long-term creep behavior without thermal disturbance, and thus serves as the baseline for evaluating accelerated methods.

### 2.3. Stepped Isothermal Method (SIM) (ASTM D6992)

The SIM, standardized in ASTM D6992, was employed to obtain accelerated creep data by applying sequential temperature increments under a constant tensile load. The procedure was clarified as follows: (i) a single specimen was mounted on the SIM apparatus and subjected to a constant load corresponding to 40–60% of its UTS; (ii) the test began at a reference temperature of 20 °C; (iii) after a dwell period of 12,000 s, during which creep strain is continuously measured, the temperature is increased in 7 °C steps (i.e., 20 → 27 → 34 → 41 → 48 → 55 °C); (iv) creep strain is recorded throughout at each step; (v) the procedure continued until rupture or a total duration of 72,000 s; and (vi) the creep data obtained at each temperature step are then vertically and horizontally shifted based on the time–temperature superposition (TTS) principle to construct a creep master curve at the reference temperature (20 °C).

A significant advantage of SIM is that one specimen provides the full temperature-dependent response, thereby minimizing specimen-to-specimen variability inherent in conventional creep testing. However, accumulated thermal strain and nonlinear viscoelastic behavior must be considered when shifting master curves for HDPE geogrids. The SIM equipment is shown in Figure 2b.

## 3. Results and Discussion

### 3.1. Conventional Creep Test Results

**Figure 3:**
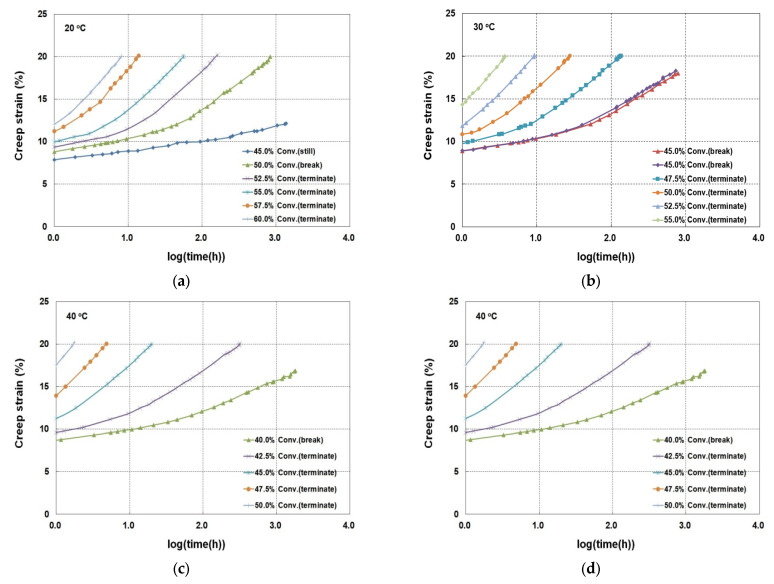
Creep strain–log(time) relationships obtained from the conventional creep tests performed at (**a**) 20 °C, (**b**) 30 °C, (**c**) 40 °C, and (**d**) 50 °C.

Previous investigations in this product range revealed that specimens subjected to creep loading exhibited diverse creep strain responses, often exceeding the design limits commonly applied in geotechnical engineering [6,7,9,10,11,32]. Accordingly, a previously established protocol was adopted, in which the tests were terminated upon reaching 20% creep strain, or earlier in the event of sample rupture. Among the 21 specimens analyzed, five ruptured shortly before reaching 20% strain, while 16 were evaluated up to the defined limit. According to ISO 13431 and ISO TS 20432, the service temperature for geosynthetics is 20 °C. Additionally, the conventional creep test results obtained at 30, 40, and 50 °C were subjected to block-shift analysis to predict the long-term creep behavior. The conventional test results at 20 °C are summarized in Table 2, while creep strain–log(time) relationships at various temperatures are presented in Figure 3.

### 3.2. Accelerated Creep Test Results

SIM master curves were obtained at six levels of applied load ranging from 40% and 60% of the UTS. The corresponding results are summarized in Table 3. As indicated, creep rupture began to occur at an applied load of 50%, and the creep strains at rupture ranged from 28.7% to 53.8% under applied loads of 50–60%.

**Figure 4:**
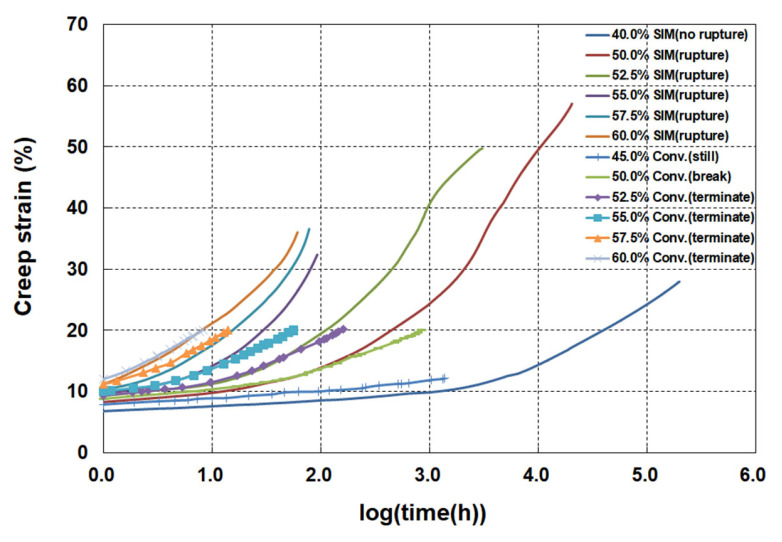
Comparison of the creep strain–log(time) curves obtained from the conventional and SIM tests.

**Figure 5:**
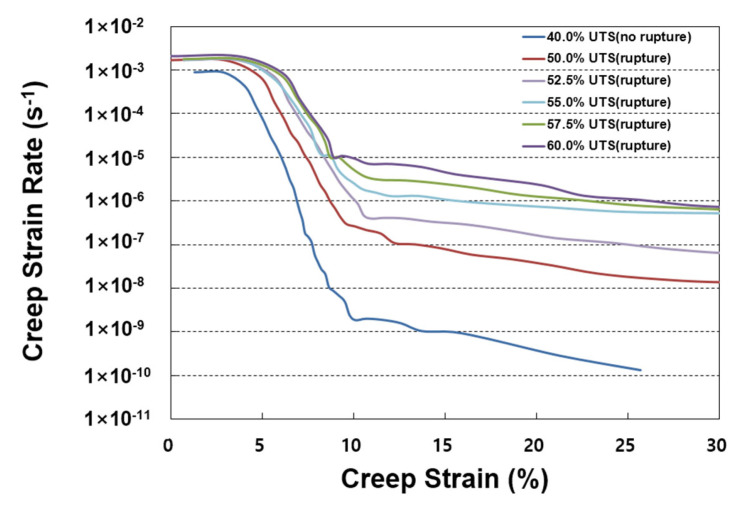
Sherby–Dorn plots obtained using the SIM results.

As presented in Figure 4, the SIM master curves were compared with the conventional creep test data obtained at 20 °C. The SIM master curves agreed well with the long-term conventional creep data in the early stage, but showed higher creep strains beyond approximately 1000 h and above 15% strain. This deviation is likely due to the accumulated strain produced during the sequential temperature steps applied in SIM, coupled with the nonlinear viscoelastic behavior characteristic of HDPE geogrids [15,27,29,35]. As shown in Figure 5, the Sherby–Dorn plots show a constant strain rate region at strains of 15–30%, indicating the onset of secondary creep. This behavior reflects the nonlinear viscoelastic response of the material, where deformation is influenced by complex interactions between increasing temperature and applied load.

To ensure consistent and meaningful creep evaluation, the creep tests in this study were conducted within 40–60% of UTS. This range corresponds to the load region identified in previous studies, where creep rupture predominantly occurs. It represents the practical domain for evaluating the long-term creep behavior of HDPE geogrids. The master curves generated under these load conditions showed good agreement between the conventional creep and SIM results in the early stage, as both methods capture the linear–viscoelastic creep response at low strain levels. However, deviations became apparent at later times, which can be explained by the nonlinear viscoelastic and thermal strain effects activated during the temperature-stepping process of SIM. These temperature-induced accelerations promote strain development relative to the conventional creep tests, resulting in divergence between the two methods in the higher-strain, long-term region. Accordingly, the RF_CR_ values were estimated using two failure criteria—20% creep strain and creep rupture—to evaluate the long-term performance reflecting the creep behavior stages of HDPE geogrids.

### 3.3. Estimation of Creep Reduction Factors

To estimate the RF_CR_ values, the failure times required to reach 20% creep strain were derived from the creep master curves under applied loads greater than 40% of the UTS. Below this loading level, the creep failure time required to reach 20% strain became excessively long, imposing practical time limitations on reliable measurement. For a 100-year design life, the RF_CR_ values based on 20% creep strain were found to be 2.90 and 3.06 using the conventional creep tests and SIM tests, respectively. The validity of these RF_CR_ values was confirmed by the fact that the differences between the values obtained from the two methods remained below 0.15 at 2000 and 10,000 h. The reliability of SIM for geosynthetic creep evaluation is supported by several publications [22,23,25,26,27,28,29,30].

The creep rupture criterion was selected as the strength limit in accordance with ISO TS 20432. In contrast, the serviceability criterion of 20% creep strain was established based on the transition in creep behavior of HDPE geogrids [1,2,11]. Strains above 20% were excluded, as most specimens did not reach these levels and reliable data would require impractically long testing times. Such high strains lie within the nonlinear/tertiary creep region, and available data were insufficient for reliable RF_CR_ estimation.

**Figure 6:**
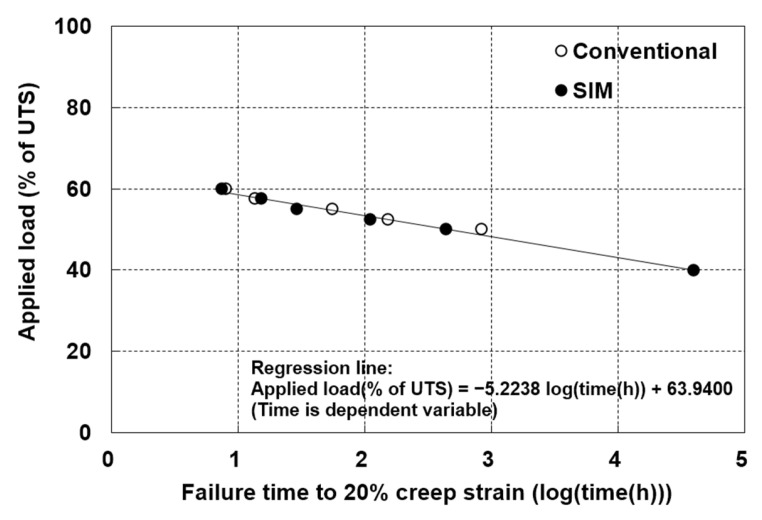
Relationship between the logarithm of the failure time and the applied load, as obtained from the combined conventional (open circles) and SIM (solid circles) data.

Subsequently, creep test data from both methods were combined to estimate RF_CR_. Assuming a linear relationship between the applied load and the logarithm of the time to failure, the linear regression lines and their corresponding equations are presented in Figure 6. Using these regression equations, the creep failure reduction factors were estimated at design lives of 50, 100, and 114 years, and these are summarized in Table 4. As shown in Figure 6 and Table 2 and Table 3, the creep failure times required to reach 20% creep strain uniformly ranged from 8 to 68,549 h. The RF_CR_ based on 20% creep strain at 100 years was determined to be 3.04 using the failure times from the conventional and SIM methods, which is comparable to the maximum value reported by Koerner [1]. This suggests that a creep strain of 20% can represent a reasonable failure criterion for HDPE geogrids used for long-term reinforcement.

### 3.4. Creep Analysis Using the TTS-Based Block Shift Method

**Figure 7:**
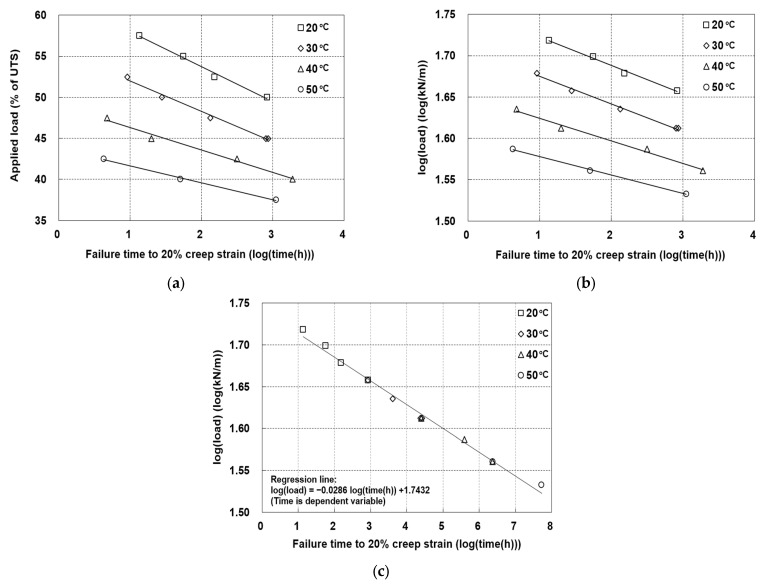
(**a**) Log(time)–applied load relationships, (**b**) log(time)–log(load) relationships, and (**c**) log(time)–log(load) relationships after block shifting. All relationships were evaluated at various temperatures under 20% creep strain.

Based on conventional creep tests conducted under various temperature and creep load conditions, the failure times required to reach 20% creep strain were calculated. The relationships between the logarithm of the failure time and the creep load are presented in Figure 7a. These test results were applied to predict the creep strength using block shifting under the TTS principle. Specifically, two consistent creep load levels were applied at each temperature to enable superposition. As shown, the data lie along a set of lines wherein the slopes are significantly steeper at higher temperatures than at lower temperatures. Since this behavior is unfavorable for block-shifting analysis, the load axis was converted to a logarithmic scale, as shown in Figure 7b. As a result, the line plots of the datasets at different temperatures became nearly parallel, allowing the block shift method to be appropriately applied. From the data presented in Figure 7b, it can be seen that the two lowest loads at temperatures of 20, 30, and 40 °C are duplicated at the next higher temperature. With this data overlap, it is possible to block each dataset to an optimum position relative to the set above without applying any mathematical construct.

The data after block shifting are presented in Figure 7c, wherein it can be seen that the data overlaps allow for the accurate positioning of each dataset relative to the one above. From the regression line of the data shown in Figure 7c, the creep strength of the tested product was calculated as 41.1% of its UTS. Moreover, from Figure 7c and Table 5, the RF_CR_ value based on a 20% creep strain at a 100-year design life was determined to be 2.43, which is lower than the value of 3.04 obtained from conventional and SIM testing.

### 3.5. RF_CR_ Based on Creep Rupture

**Figure 8:**
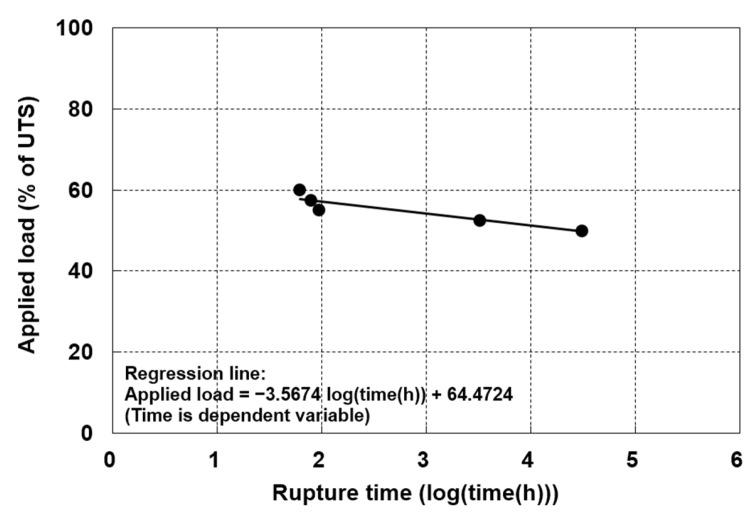
Rupture diagram based on the SIM data obtained for the HDPE geogrid.

Because creep rupture times are difficult to obtain through conventional creep testing, the RF_CR_ values based on creep rupture were estimated exclusively using SIM data. The creep rupture reduction factor adopted as the failure criterion in ISO TR 20432 was employed for the geogrids evaluated in this study. The linear relationship between the applied load and the logarithmic creep rupture time is presented in Figure 8. Five rupture times were plotted, including three between 1 and 1000 h, one between 1000 and 10,000 h, and one beyond 10,000 h. The RF_CR_ values were estimated for design lives of 50, 100, and 114 years using the regression equation, and these are summarized in Table 6. Consequently, the estimated creep rupture reduction factor was determined to be 2.30 at 100 years, corresponding to 43.5% of the applied load. Using the SIM, five creep rupture times were obtained. However, under the SIM test conditions, rupture was observed only within the load range of 50–60%, with rupture times spanning from 7 to 39,537 h. This limitation indicates that the dataset does not provide uniformly distributed rupture times, thereby constraining the robustness of creep reduction factor calculations. Nonetheless, the observed linear relationship between log(load) and log(time-to-rupture) is consistent with established findings in the literature [1,22].

The rupture-based RF_CR_ (2.30) was lower than the strain-based RF_CR_ values, and this difference can be attributed to the fundamental distinction between the two failure mechanisms. The 20% creep strain criterion represents a serviceability-based limit and marks the onset of significant nonlinear creep in HDPE geogrids. In contrast, creep rupture is a strength-based limit that occurs only after the material enters the tertiary creep region. In this region, deformation accelerates rapidly due to chain disentanglement, lamellar fragmentation, and temperature-activated viscoelastic softening. Because serviceability failure occurs earlier and at lower effective deformation levels, the RF_CR_ derived from the 20% strain criterion is inherently more conservative than the rupture-based RF_CR_. This explains why the rupture-based RF_CR_ (2.30) does not match the higher RF_CR_ values obtained from the strain-based criterion.

## 4. Conclusions

This study evaluated the long-term creep behavior of HDPE geogrids using conventional creep testing (ISO 13431), the stepped isothermal method (ASTM D6992), and TTS-based block-shift analysis. The main findings are as follows:

(i) Agreement Between SIM and Conventional Creep

SIM master curves matched the conventional creep data in the early stage, confirming that SIM accurately captures the linear–viscoelastic response. At strains above ~15%, SIM predicted higher creep due to temperature-induced nonlinear viscoelasticity and accumulated thermal strain.

(ii) Secondary Creep Confirmation

Sherby–Dorn plots showed a constant strain-rate region at 15–30% strain, indicating the onset of secondary creep and explaining the divergence between SIM and conventional results at longer durations.

(iii) RF_CR_ Based on 20% Creep Strain

For a 100-year design life, the RF_CR_ values were 2.90 (conventional) and 3.06 (SIM), with differences below 0.15 at 2000 and 10,000 h. This agreement supports the use of SIM for estimating strain-based RF_CR_. Furthermore, block-shift analysis yielded an RF_CR_ of 2.43 at a 20% creep strain criterion for the 100-year design life.

(iv) RF_CR_ Based on Creep Rupture

Rupture-based RF_CR_ values provided complementary results and were more conservative at high loads due to the accelerated transition to tertiary creep.

(v) Rationale for Failure Criteria

While the creep rupture criterion specified in ISO TS 20432 provides a general framework, it has practical limitations when applied to HDPE geogrids. This study therefore proposes a 20% creep strain criterion as a reliable and conservative basis for calculating RF_CR_, as it reflects the transition in viscoelastic–viscoplastic creep behavior. Creep strains exceeding 20% exhibit nonlinear/tertiary creep behavior and are therefore not considered as a creep failure criterion.

(vi) Applicability of SIM

SIM is suitable for characterizing long-term creep behavior within the linear and early nonlinear regimes but must be interpreted cautiously at high strain levels.

(vii) Engineering Implications

The dual-criteria RF_CR_ approach enhances the reliability of long-term design predictions and offers practical guidance for selecting RF_CR_ values for HDPE geogrids in reinforced-soil structures with service lives of 50–100 years.

## Figures and Tables

**Table 1:** Tensile properties of the HDPE geogrid used in this work.

	Measured Tensile Strength (kN/m)	Measured Elongation at Maximum Load (%)
Mean	95.2	10.8
Standard deviation	0.8	0.4

**Table 2:** Summary of the conventional test results at 20 °C.

Creep load (% of UTS)	50.0	52.5	55.0	57.5	60.0
Failure time (h) at 20% creep strain	-	152.4	55.7	13.6	8.0
Rupture time (h)	835.0	-	-	-	-
Rupture strain (%)	19.6	-	-	-	-

**Table 3:** Summary of the SIM test results.

Creep load (% of UTS)	40	50	52.5	55	57.5	60
Failure time (h) at 20% creep strain	39,537	443	110	29	15	7.5
Rupture time (h)	-	30,479	3258	94	78	62
Rupture strain (%)	-	53.8	45.6	28.7	32.8	32.6

**Table 4:** Summary of the creep reduction factors (RF_CR_) calculated from the conventional and SIM test results at 20 °C based on a 20% creep strain.

Design life (years)	50	100	114
RF_CR_ (using the conventional test at 20 °C)	2.78	2.90	2.92
RF_CR_ (using the SIM test at 20 °C)	2.92	3.06	3.09
RF_CR_ (using a combination of the conventional and SIM tests)	2.90	3.04	3.07

**Table 5:** Creep reduction factors (RF_CR_) calculated from block-shift analysis based on a 20% creep strain.

Design life (year)	50	100	114
RF_CR_ (using block-shift analysis)	2.38	2.43	2.44

**Table 6:** Creep reduction factors (RF_CR_) calculated from the SIM results based on creep rupture.

Design life (year)	50	100	114
RF_CR_ (using block-shift analysis)	2.24	2.30	2.31

## Data Availability

The original contributions presented in this study are included in this article. Further inquiries can be directed to the corresponding authors.

## Abbreviations

The following abbreviations are used in this manuscript:

GRS	Geosynthetic reinforced soil
HDPE	High-density polyethylene
PET	Polyethylene terephthalate
RF_CR_	Creep reduction factor
SIM	Stepped isothermal method
T_g_	Glass transition temperature
T_m_	Melting temperature
TTS	Time–temperature superposition
UTS	Ultimate tensile strength
